# Now is the time to introduce new innovative assisted reproduction methods to implement accessible, affordable, and demonstrably successful advanced infertility services in resource-poor countries

**DOI:** 10.1093/hropen/hoaf001

**Published:** 2025-01-17

**Authors:** Willem Ombelet, Jonathan Van Blerkom, Gerhard Boshoff, Carin Huyser, Federica Lopes, Geeta Nargund, Hassan Sallam, Koen Vanmechelen, Rudi Campo

**Affiliations:** The Walking Egg Non-Profit Organization, Genk, Belgium; Faculty of Medicine and Life Sciences, LCRC, University of Hasselt, Diepenbeek, Belgium; Department of Molecular, Cellular and Developmental Biology, University of Colorado, Boulder, CO, USA; Faculty of Medicine and Life Sciences, LCRC, University of Hasselt, Diepenbeek, Belgium; Department of Obstetrics and Gynaecology, University of Pretoria, Pretoria, South Africa; Department of Obstetrics and Gynaecology, University of Pretoria, Pretoria, South Africa; School of Medicine, University of Dundee, Dundee, UK; St George’s Hospital NHS Trust, London, UK; Department of Reproductive Medicine, Alexandria University Faculty of Medicine, Alexandria, Egypt; The Walking Egg Non-Profit Organization, Genk, Belgium; The Walking Egg Non-Profit Organization, Genk, Belgium; Life Expert Centre, Leuven, Belgium

**Keywords:** accessible, affordable, assisted reproduction, infertility care, LMICs, low- and middle-income countries, simplified IVF

## Abstract

Nearly 200 million people worldwide suffer from infertility. Disparities exist between developed and developing countries due to differences in the availability of infertility care, different reimbursement policies and socio-cultural differences surrounding procreation. In low- and middle-income countries, specialized infertility centres are either scarce or non-existent, mostly in private settings, and accessible only to the fortunate few who can afford them. The success and sustainability of ARTs will depend on our ability to optimize these techniques in terms of availability, affordability, and effectiveness. A low-cost, simplified IVF system has been developed and shown to be safe, cost-effective, and widely applicable to low-resource settings. Combined with inexpensive mild ovarian stimulation protocols, this could become a truly effective means of treating infertility and performing assisted reproduction at affordable prices, but only if such programmes are sincerely desired and supported by all relevant stakeholders. A receptive political, governmental, and clinical community is essential.

## Introduction

Infertility is one of the most common chronic diseases in individuals of reproductive age, affecting roughly 8–12% of populations worldwide (Boivin *et al.*, 2007). It is estimated that approximately one in every six individuals of reproductive age worldwide is affected by infertility (World Health Organization, 2023). Collective estimates from a comprehensive systematic review of lifetime and period prevalence of 12-month infertility were 17.5% and 12.6%, respectively (Cox *et al.*, 2022). By using a different methodological approach, Mascarenhas *et al.* (2012) calculated that 70 million couples worldwide will require some degree of medical assistance to achieve pregnancy. The prevalence of infertility appears to be higher in low- and middle-income countries (LMICs), with rates as high as 30–40% reported in some regions of sub-Saharan Africa (Ombelet *et al.*, 2008; Inhorn and Patrizio, 2015; Polis *et al.*, 2017; Legese *et al.*, 2023).

Infertility is often most prevalent in regions with high fertility rates, a demographic paradox known as ‘barrenness amid plenty’. The United States Agency for International Development (USAID) estimated that there are more than 200 million women and girls with unmet needs for contraception each year. Unmet need is defined for women who want to delay or stop childbearing. Particular challenges regarding contraception include lack of access due to the absence of appropriate health services, fear of side effects, fewer method options, and ‘stock-outs’ of contraceptive supplies (Schivone and Blumenthal, 2016). For adolescent African girls in particular, this partly explains why fertility levels are high, especially in rural areas.

Although primary infertility is generally known to have the highest burden of disease, secondary infertility also applies to many who have become pregnant but have subsequently experienced a pregnancy loss or death of a child (Inhorn and Patrizio, 2015). Some studies have reported significantly higher rates of secondary infertility, compared with primary infertility, in certain regions such as Africa, where rates of infection-related infertility from postpartum infections or unsafe abortions are high (Larsen, 2000; Sharma *et al.*, 2009).

The impact of infertility and unintended childlessness in LMICs tends to be much more pronounced than in Western societies, particularly for women. In these fundamentally pronatalist contexts, childless women are often stigmatized, isolated, ostracized, disinherited, and neglected by the whole family and not infrequently, by their local community. This can lead to polygamy, divorce, intimate partner violence (IPV), isolation, economic instability, banishment, and even suicide in some cases (Dyer *et al.*, 2002, 2004, 2005; Ombelet *et al.*, 2008; Inhorn and Patrizio, 2015; Chiware *et al.*, 2021; Wang *et al.*, 2022). In many LMIC societies, women are completely dependent on children for economic survival. The birth of a child can secure a marriage, guarantee property and inheritance rights, and/or provide social security in old age (Dyer and Patel, 2012; Editorial, The Lancet Global Health, 2022). Childlessness must therefore be seen as a social and public health issue, additional to the individual medical problem, and treated as such with compassion and dignity.

Despite the severe burden associated with childlessness in most LMICs and notwithstanding the recognition, concerns, and often recommendations expressed by national and international health organizations and private humanitarian and philanthropic programs, barriers to infertility care persist and thus it remains a low priority for local politicians, community leaders, and healthcare providers (Ombelet *et al.*, 2008; Chiware *et al.*, 2021; Gerrits *et al.*, 2023; Whittaker et *al.*, 2024). Typically, the absence of affordable fertility services in LMICs has been justified by arguments of overpopulation and limited resources, resulting in inequitable access to infertility treatment compared to developed countries (Ombelet *et al.*, 2008).

Many articles in the most influential journals that address the problems of imbalanced access to ART in LMICs focus mainly on ‘awareness campaigns’ to prevent infertility in susceptible individuals and destigmatising of infertility. Policymakers are coaxed to increase societal funding and to create sustainable multidisciplinary and multi-stakeholder consortia to improve equity of access (Starrs *et al.*, 2018; Afferri *et al.*, 2022; Fauser *et al.*, 2024). Strangely, almost all of these articles barely discuss the essential reason for inequalities in access to ART in LMICs: the high cost. Advanced ART methods require highly skilled personnel, expensive equipment and consumables, and rigorous maintenance. Once the logistical, pecuniary competitive interests, and costs to build and operate ART programs to high-resource country standards are realized, the good intentions, apparent enthusiasm and verbal support by the aforementioned groups and local governing bodies dissipate. Although it seems self-evident, the obvious solution to eliminating these barriers and rekindle enthusiasm for expanding access to treatment for those under-served is to reduce logistical and operating costs. While this solution sounds simplistic, the implementation of it is complex, although entirely feasible, as discussed in the following segment.

According to a WHO-initiated systematic landscape analysis, one of the most important limitations when considering implementation options for low-cost ART in LMICs is the lack of high-quality outcome-based trials (Chiware *et al.*, 2021). They concluded that affordable ART initiatives should be evaluated for efficacy and safety through robust research and further adapted to local infrastructure. Despite the fact that a number of high-quality studies have been published reporting on the efficacy, safety, cost-effectiveness, and successful outcomes of using a simplified, low-cost IVF system (Ombelet *et al.*, 2022a,b, 2023a,b), interest in this approach remains remarkably low among infertility specialists, societies, NGOs, foundations, and industry and healthcare managers. A 2022 Editorial in The Lancet Global Health mentioned that ESHRE called for action to give adequate attention to the issue of infertility in developing countries (Editorial, The Lancet Global Health, 2022). Indeed, in 2008, the ESHRE special task force on ‘Developing Countries and Infertility’ held an expert meeting in Arusha, Tanzania, resulting in a ‘Human Reproduction Monograph’ stating: ‘After a fascinating period of almost 30 years of IVF and 15 years of ICSI, we must admit that only a small part of the world population benefits from these new reproductive technologies. Time has come to give adequate attention to the issue of infertility in developing countries’ (Ombelet, 2008). The editor questioned why this call for action has not led to a wake-up call and a better and more effective approach to this problem over the next 15 years.

## An international call for affordable infertility care in LMICs

The right of persons to access infertility treatment is a human dignity that was confirmed in consecutive UN international statements (United Nations 1948, 2014, 2000). Article 12 of the International Covenant on Economic, Social and Cultural Rights also acknowledges the right of everyone to the highest attainable standard of physical and mental health (United Nations, 1966).

In a fact sheet published in 2021, the World Health Organization (WHO) recognizes that the provision of quality family planning services, including fertility services, is one of the core elements of reproductive health (World Health Organization, 2021, 2024). This very important message includes a necessary requirement and desire to work with interested and relevant stakeholders to provide fertility services globally and to provide technical assistance at a country level to Member States in order to develop or strengthen the implementation of national fertility policies and services.

Although addressing infertility is fundamental to realizing the right of individuals and couples to establish a family (Mburu *et al.*, 2023), political declarations and commitments need to be followed by action, and progress towards these goals still remains virtually non-existent. Important international non-profit organizations (NPOs) including Family Health International, International Planned Parenthood Federation and The Population Council still focus primarily on the prevention of unwanted pregnancies, safe motherhood, and the reduction of unsafe abortions, as well as the prevention of sexually transmitted infections (STIs) and HIV/AIDS. Even today, the implementation of affordable infertility care in LMICs is not a priority for these organizations despite the fact that this message is increasingly cited in the most influential scientific journals (Vayena *et al.*, 2009; Murage *et al.*, 2011; Hammarberg and Kirkman, 2013; Inhorn and Patrizio, 2015; Starrs *et al.*, 2018; Chiware *et al.*, 2021; Fauser *et al.*, 2024).

## Causes of infertility in LMICs

Infertility may be caused by female and/or male factors or may remain unexplained. Female factors include advanced reproductive age and the resultant diminished ovarian reserve, chronic anovulation, and tubal factor infertility or other pelvic pathologies such as endometriosis, adenomyosis, and uterine congenital anomalies. Male infertility can result from impaired sperm production due to a variety of underlying conditions including hormonal, infectious, genetic, and environmental aetiologies (World Health Organization, 2023).

The causes of infertility vary according to country/region. In many LMICs, from Asia to Latin America and Africa, infection-related tubal blockages are an important cause of female infertility as a result of poor obstetric and postpartum care, untreated STIs, unsafe abortions, and harmful cultural practices (Ombelet *et al.*, 2008; Ombelet and Onofre, 2019).

Particularly in sub-Saharan Africa, STIs are the most common cause of female and male infertility. As tubal factor infertility and severe male infertility are best treated with expensive IVF-related procedures, we should be aware that the most expensive form of treatment is usually what is needed in the majority of cases in the poorest countries.

## Lack of affordable infertility care in LMICs

Treatment of infertility is generally not prioritized in national population and development policies or reproductive health strategies of LMICs and is rarely covered by public health financing (Afferri *et al.*, 2022). Severe or life-threatening conditions rightly take precedence over expensive fertility treatments. However, these priorities fail to recognize the severe psychosocial and economic burden of infertility in LMICs (Inhorn and Patrizio, 2015; Chiware *et al.*, 2021) and the options for providing such care at an affordable cost.

Access to healthcare is disease-specific and is determined by both the demand for and supply of such services. Affordability to consumers is a strong driver for access. Affordability can be changed by: (1) reducing the cost and complexity of infertility interventions, (2) providing reimbursement policies, and (3) increasing the disposable income of individuals. Currently, it is apparent that while only the cost of treatment and reimbursement policies are amenable to policy intervention, the implementation of innovations that can expand access and reduce inherent costs remains inadequate.

On average, ART costs range between 10 000 and 20 000 USD in the USA and between 3000 and 6000 USD in most LMICs, with substantial variations between and within countries (Njagi *et al.*, 2023). These costs may be direct or indirect; according to Huyser and Boyd (2013), one-third of these direct costs are linked to laboratory expenses and almost one-third are explained by medication costs. Indirect cost includes costs due to complications (multiple pregnancies, thrombo-embolic diseases, ovarian hyperstimulation syndrome, etc.) and can be considerable as they are typically borne by the community as a whole.

To meet a population’s need for ART, it has been estimated that, annually, at least 1500 couples per million inhabitants should have access to IVF (Fauser *et al.*, 2002). This is only possible if adequate ART centres are available, and the costs associated with ART treatment are within reasonable limits, which is surely not the case in LMICs (Makuch and Bahamondes, 2012; Ombelet and Onofre, 2019; Chiware *et al.*, 2021; Afferri *et al.*, 2022).

## Introduction of low-cost initiatives for LMICs

To achieve universal access to ART, it is crucial to develop low-cost ART models with simplified diagnosis and treatment protocols, while maintaining quality of care. What are the barriers to making ART less expensive? ART is a booming business; the global market was valued $34.7 billion in 2023 and is expected to reach $62.8 billion in just 10 years (Editorial, The Lancet, 2024). This growth is driven by increasing demand for fertility treatments, ‘add-on’ treatments and advancements in reproductive technologies such as preimplantation genetic testing for aneuploidy and monogenic mutations. However, while the ‘fertility enterprise’ continues to grow, access to IVF remains limited or non-existent for most of the world’s population because of high treatment costs. In more than half of LMICs, the direct cost for one ART cycle is higher than the average annual gross domestic product per capita (Njagi *et al.*, 2023). Better access to effective and safe patient-centred and evidence-based treatments is needed. A profit-driven fertility industry cannot continue to exploit the vulnerability of people desperate to have children (Editorial, The Lancet, 2024). Therefore, it is evident that low-cost initiatives are urgently needed to meet the demand.

## Non-IVF treatment options

In the era of IVF, we sometimes overlook many other infertility treatment strategies. Ovulatory dysfunction represents almost 20% of female infertility and can be treated effectively with a low-dose ovarian stimulation regimen using clomiphene citrate and/or gonadotrophins combined with timed intercourse. In sub-fertile women with anovulatory polycystic ovary syndrome (PCOS), letrozole or laparoscopic ovarian drilling is recommended (Franik *et al.*, 2022). For women younger than 40 years with unexplained infertility or moderate male infertility, three to six cycles of IUI with mild ovarian stimulation should be recommended as a first-line therapy, provided tubal patency has been documented and a strict cancellation strategy is followed to avoid multiple pregnancies (Cohlen *et al.*, 2018). Endoscopic surgery can be a valuable and cost-effective solution in case of moderate and severe endometriosis, uterine malformations, PCOS, intrauterine adhesions, etc. Although these treatment options are very valuable, a large proportion of the infertile population will ultimately only be assisted through IVF-related techniques. For these patients, low-cost ART services are needed and can be largely achieved with a lower price tag achieved by using mild ovarian stimulation protocols and simplified IVF laboratory design and procedures.

## Implementation of simplified low-cost ART services

### Mild ovarian stimulation

The value and effectiveness of less expensive and less intensive mild ovarian stimulation protocols in ART settings have been proven (Nargund and Fauser, 2020; Nargund *et al.*, 2022). The use of clomiphene citrate or tamoxifen with or without low-dose recombinant FSH or hMG (gonadotrophins) can be an affordable alternative with acceptable results in all categories of women compared to conventional stimulation IVF, with the added benefit of minimal side effects and a very low complication rate (Ferraretti *et al.*, 2015; Datta *et al.*, 2020, 2021; Gianaroli *et al.*, 2022; Nargund *et al.*, 2022). Beside harm minimization, this approach also reduces the cost for the public health system.

### Simplified IVF laboratory procedures

**Figure 1.**
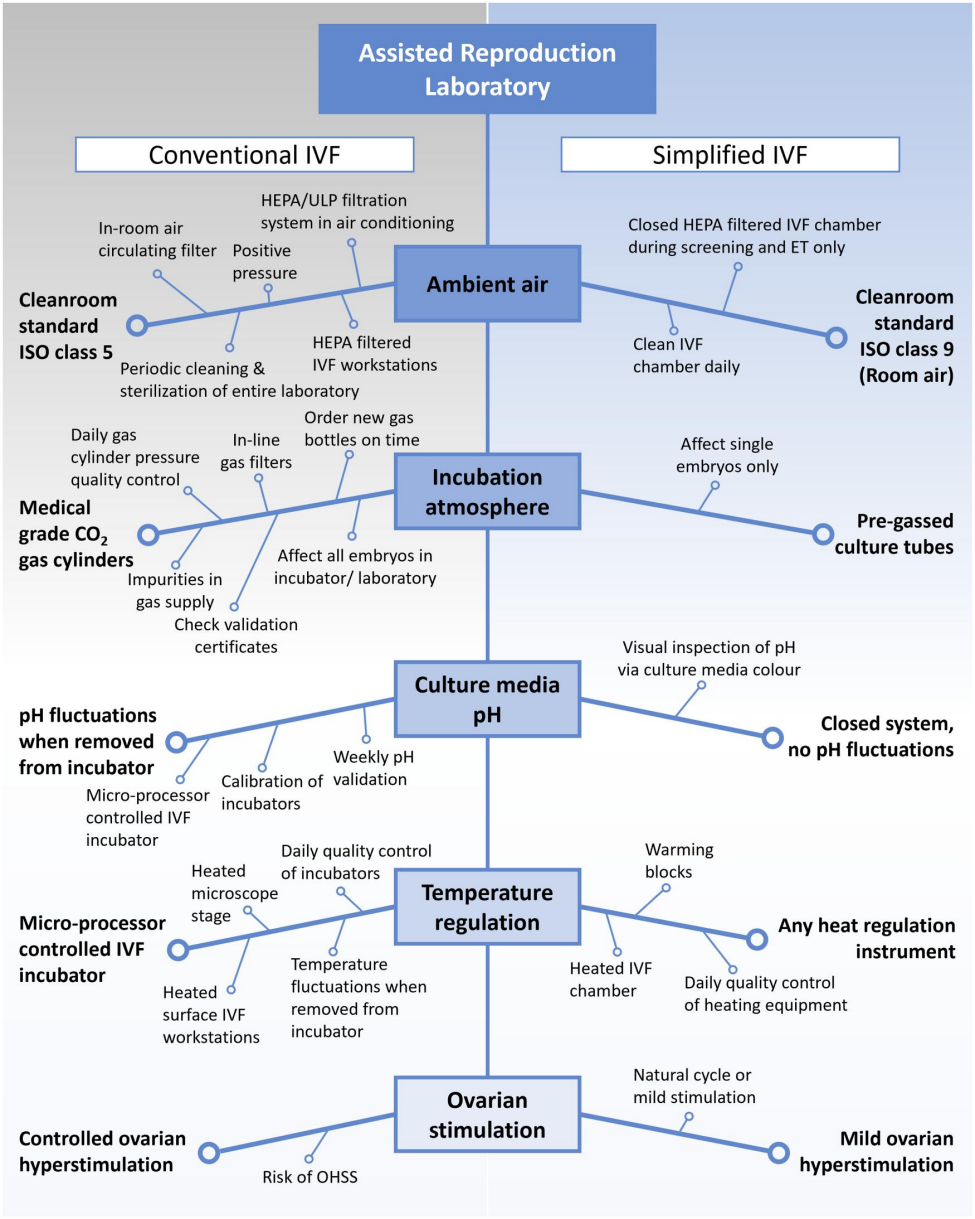
A comparison of the simplified culture system with conventional IVF culture, showing various required parameters that need to be controlled.

In 2014, we published the results of using a novel simplified IVF culture system (SCS) (Van Blerkom *et al.*, 2014). This system avoids logistical issues common in high-complexity programs (e.g. supply chain disruptions and on-time delivery of disposables such as culture vessels and media), the high costs of medical gases, and the complex incubation equipment and infrastructure typical of IVF laboratories in high-resource settings (Fig. 1). IVF insemination was also addressed whereby only 2000–5000 motile washed spermatozoa showed successful outcomes with virtually no fertilization abnormalities such as dispermic penetration, which means this system can be used in cases of mild and moderate male infertility in lieu of ICSI with similar outcomes (Van Blerkom *et al.*, 2014; Ombelet *et al.*, 2022b). In the case of severe male infertility where too few motile and morphologically normal spermatozoa are obtained after processing for conventional IVF, ICSI would likely be appropriate in these specific instances. In the simplified system, preimplantation embryogenesis from insemination to transfer or for cryopreservation, is undisturbed and occurs within the same culture tube. This avoids many of the problems often encountered in regular IVF laboratories, such as unwanted temperature changes, air quality problems, as well as prolonged examination for fertilization and stage-appropriate developmental progression, especially where costly time-lapse incubation systems are unavailable or affordable.

With the combination of mild ovarian stimulation, fewer embryos are expected, but even so, there would be excess embryos from time to time. Cryopreservation of embryos has become both commonplace and a cost-effective necessity that can improve the likelihood of a successful outcome in infertility treatments. While we have demonstrated that successful outcomes from cryopreservation can be achieved using the SCS approach (Ombelet *et al.*, 2014), its use in an SCS/WE program might seem counterintuitive for low costs and a significant negative factor for adoption. This would be a relevant concern if the controlled rate freezing with expensive programmable instrumentation was still in use. By contrast, vitrification with demonstrably higher success rates has significantly simplified the cryopreservation process in terms of materials, laboratory personal training, time and effort, and with respect to outcomes, it is a cost-effective option when a fresh embryo transfer fails.

We recently reported the results of a prospective non-inferiority study comparing ICSI and SCS using sibling oocytes. No differences in ongoing pregnancy rate, implantation rate, and miscarriage rate between SCS and ICSI were detected in investigating 653 SCS/ICSI cycles (Ombelet *et al.*, 2022b). In the same patient cohort, we observed similar perinatal outcomes for babies born after SCS and ICSI, both in fresh and frozen-thawed cycles (Ombelet *et al.*, 2022a). In another study, it was shown that for SCS singletons, the preterm birth (PTB) and low birthweight (LBW) rates were significantly lower compared to a large cohort of all babies born after conventional IVF in Belgium during the same study period (Ombelet *et al.*, 2023b). When comparing the PTB and LBW rates of the SCS singletons with all 553 865 singletons conceived and born in Flanders, the PTB and LBW rates were found to be similar to those of singletons born after natural conception. One potentially important finding was that compared to babies born after ovarian stimulation and IVF/ICSI, SCS singletons had lower PTB and LBW rates (Ombelet *et al.*, 2023a). These studies provide sufficient evidence that the SCS technology is a safe, effective, and successful low-cost method and well-suited for the purpose for which it was designed: implementation in low- and moderate-resource settings where most infertile couples reside.

From a cost–benefit point of view, the differences in costs when setting-up, managing and maintaining a conventional high-tech IVF laboratory versus a SCS laboratory were examined. The discounted cash flow method (DCF) was chosen to evaluate the investments. The results showed that the SCS laboratory clearly presented the highest net present value (NPV) and was identified as the most attractive investment for its purpose (Christiaens, 2018).

In the Science Museum in London, and on the occasion of the celebration of 40 years of IVF in 2018, an exhibition called ‘IVF*: Six Million Babies Later*’ was opened where visitors can see the equipment used in the first human IVF lab alongside the new ‘shoebox’ or SCS culture and incubator equipment ‘designed to dramatically reduce the cost and improve the accessibility of IVF’. The two culture systems are fundamentally similar, as the new low-cost system uses the combination of a simplistic back-to-basics approach in regards to disposables and protocols, and 40 years of experience and improvements in culture media and knowledge of important steps and quality control measures. The results with the SCS system are similar to those reported by contemporary high-resource IVF programs, indicating that costly and complex instrumentation is not always required to achieve successful outcomes; nor does the location of the IVF laboratory need to be fixed.

**Figure 2.**
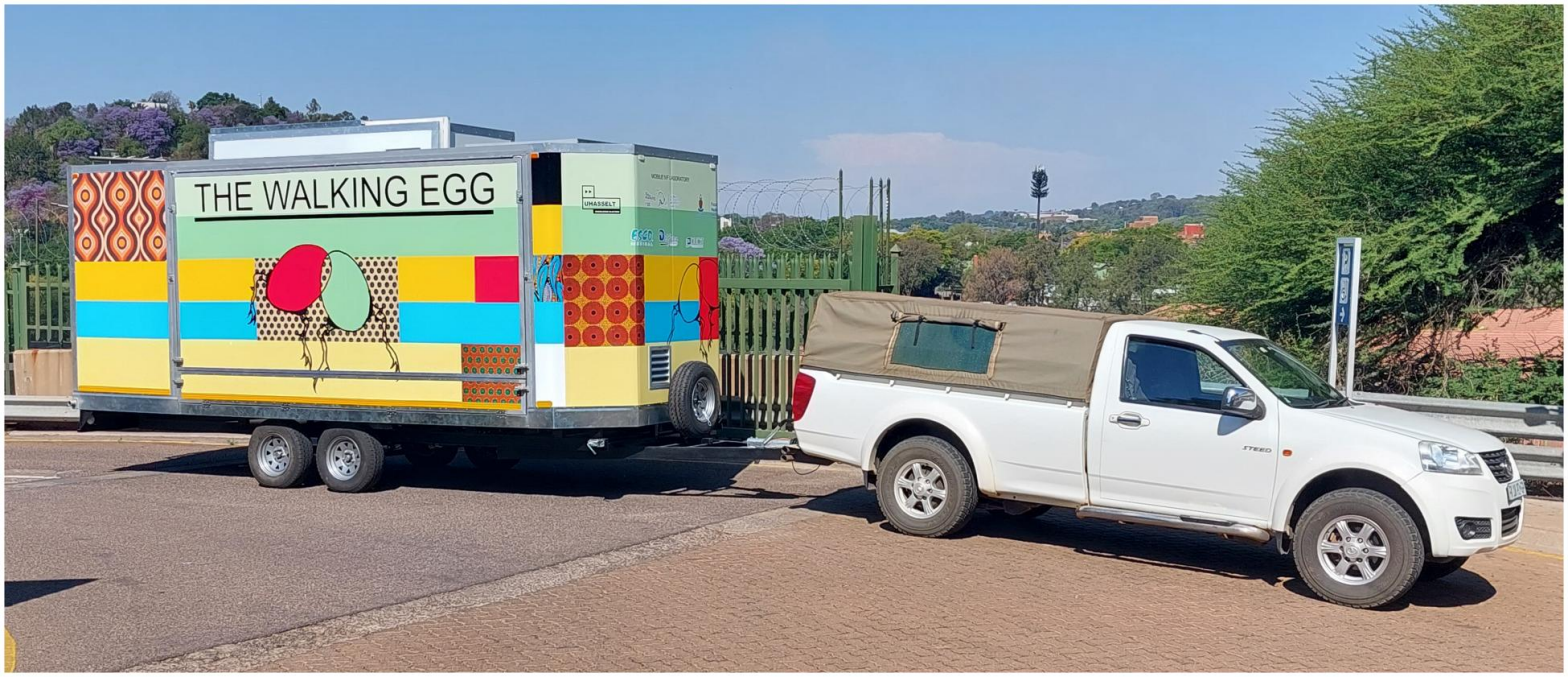
First performance and show of The Walking Egg mobile IVF unit in Pretoria, South Africa on 28 October 2023.

In October 2023, the first mobile unit for the Walking Egg Project (Dhont, 2011; Ombelet, 2014) was unveiled in Pretoria, South Africa (Fig. 2). The Walking Egg non-profit organization was founded in 2010 with the aim of raising awareness of childlessness in resource-poor countries and making infertility treatment in all its aspects, including ARTs, available and accessible to a much larger portion of the population. The mobile unit, as it was shown in Pretoria, contains all the equipment needed for simplified IVF, including an embryo transfer room. The mobile unit was designed to comply with all necessary quality requirements of an IVF laboratory, ensuring conventional IVF standard culture provided in a different manner (Fig. 1). In combination with a facility providing a procedure room for oocyte retrieval, the mobile unit can perform most ART procedures, including semen analyses and processing with cryopreservation, IUIs, oocyte retrievals with cryopreservation or IVF insemination, and embryo culture followed by embryo transfer or cryopreservation, as well as subsequent embryo thawing and transfer.

The conversion to mobile laboratories would enable a larger population to access ART services and provide these services to economically disadvantaged infertile couples in resource-poor communities or regions without specialized infertility centres. Prospective studies investigating mobile IVF outcomes are currently on-going.

### Training and data collection

Training, quality control, regular audit, and systems of accreditation and registration need to be in place to maintain appropriate standards of care and external verification of outcomes, as is generally practiced in IVF programs, as noted below. Training will need to be supported by experts in the field who are to deliver appropriate courses at the highest level in a very short period of time. While the levels of experience of trainees, the quality of facilities, and health policies and regulations can be country-specific, we do not consider this to be a barrier to adoption as much can be effectively done in a preliminary manner online with in-person follow-up prior to patient implementation of the SCS. In this context, while the implementation of low-cost infertility treatment in LMICs may sound daunting, we have found, for the SCS, that with a reasonable training program specifically designed for intended embryologists, the necessary experience and expertise can be attained. Training will not only involve laboratory personnel but also medical, paramedical, and administrative members of the IVF team. An effective online capacity can be available such that issues associated with oocyte quality, fertilization, or embryo development can be viewed and discussed remotely with members of the Walking Egg Program (Van Blerkom *et al*, 2019), so no centre implementing this system can be without external expertise if and when needed.

Given the lack of access to national ART data in most LMICs, we suggest that for centres providing low-cost infertility treatment, registration of all cycles in a national or international registry, preferably using an online system, is required from the outset. Regular audits and systems of accreditation and registration should be implemented to maintain appropriate standards of care.

## Declining global fertility rates and family building

The GBD 2021 Fertility and Forecasting Collaborators (2024) recently published a comprehensive demographic analysis with forecasts to 2100. They concluded that ‘by 2100, the largest concentrations of livebirths will shift to low-income settings, particularly a subset of countries and territories in sub-Saharan Africa, which are among the most vulnerable to economic and environmental challenges. Extreme shifts in the global distribution of livebirths can be partially ameliorated by improved female education and met need for modern contraception’.

This indirectly underlines the fact that infertile couples in these countries can expect little help in the future and that their problem is completely unaddressed, despite the undeniable need for reproductive justice for people suffering from infertility in LMICs. The number of babies that would be added to this population as a result of ART is minuscule, yet the suffering of people is being completely ignored.

## The need for funding and support of all main stakeholders

Infertility is a reproductive health concern deserving attention, as confirmed by the 2018 report of the Guttmacher-*Lancet* Commission on Sexual and Reproductive Health and Rights (Starrs *et al.*, 2018). Fathalla *et al.* (2006) have already argued that sexual and reproductive health for all is an achievable goal if cost-effective interventions can be properly scaled up, if political commitments are revitalized, and if financial resources are mobilized, allocated rationally and used more effectively.

All international fertility societies and organizations are nowadays appealing for action to increase access to infertility care and ART in LMICs, but governments and sexual and reproductive health rights (SRHR) organizations tend to neglect infertility. We still observe a lack of commitment or willingness to take advantage of low-cost IVF options even from the very same sources that champion women’s ‘reproductive rights’ based on ‘social justice’ (Van Blerkom *et al.*, 2019). Despite the undeniable value of prevention programmes and awareness campaigns, we believe that the most obvious and timely approach to increase accessibility to proper infertility management in LMICs is to reduce costs significantly. Soon, one cannot expect that insurance companies and governments will reimburse ART in LMICs unless we can prove that we can provide high-quality and successful infertility care at a reasonable price, with special attention to avoid complications such as multiple pregnancies and OHSS which contribute very high societal costs.

This also means that we urgently need funding to perform research on the effectiveness, safety, and costs associated with the implementation of low-cost fertility centres in LMICs.

The need for funding is crucial and is likely to require input and collaboration from various private, public, and governmental participants who, by necessity, have a central role in the implementation of this endeavour. Funding is needed for: (1) the fixed costs of establishing and operating new fertility centres and, where appropriate, mobile units, (2) training the medical, paramedical, and administrative staff, and (3) educating the public, which implies contacts with schools, politicians, traditional healers, and the media, as appropriate for each country. The most important recommendations for setting up low-cost IVF centres in LMICs are summarized in Table 1.

**Table 1.** Overview of the most important recommendations to consider when starting low-cost IVF centres in low- and middle-income countries.

**Community**
The community/region should be empowered to support the program.
Information to the community must be discrete and applicable, taking into account socio-cultural and religious differences.
The integration of family planning, safe motherhood care, and infertility services should be pursued.
**Low-cost IVF centres**
Locations for pilot-projects need to be decided.
A business plan with clear cost structures must be formulated.
**Personnel and support services**
Sufficient trained personnel must be available to reliably offer ART services without interruption.
A training programme for medical and paramedical staff should be designed if needed. This implies a careful and strict follow-up and regular audits,
Support for low-cost IVF centres must be available to have a ready supply of appropriate disposable items and medication, along with advice from experienced experts in the relevant fields.
The implementation and roll-out of a low-cost IVF centre must be planned to fall within the local framework of healthcare providers, with access to referral services for more advanced cases.
**ART protocols, equipment, and disposables**
The application of ART should be designed to be practical, repeatable, and efficient.
Equipment should be basic, purpose-made, and robust.
IVF laboratory products should be ready-to-use and should have a long shelf-life.

We sincerely hope and expect that the highly profitable medical and pharmaceutical industries that have supported IVF in high-resource countries will continue to do so by making relevant contributions, (such as providing drugs at low cost, producing basic ultrasound and laboratory equipment at low cost) and participate in the expansion of the novel mobile units described above. As the fertility industry in LMICs evolves, there is a risk that the focus will shift from evidence-based and patient-centred practice to shareholder returns and business growth. However, a for-profit fertility industry cannot continue to ignore the vulnerability of people who desperately want to have children (Editorial, The Lancet, 2024).

According to an article in the 22 July 2023 issue of The Economist, developing technologies to make IVF more affordable could potentially increase the number of IVF babies worldwide from the current 64 000 per month to more than one million per month, which would clearly be a boon to those it is intended to serve (The Economist, 2023). In May of 2024, the WHO published another factsheet on infertility. Although they mention that ‘ART technologies are still largely unavailable, inaccessible, and unaffordable in many parts of the world, particularly in LMICs’, the crucial issue of lowering the costs associated with ARTs is not mentioned at all (World Health Organization, 2024).

## Conclusions

In the majority of LMICs, access to well-organized, high-quality fertility centres is woefully lacking and, when available, is too expensive for the vast majority of the population. We need to stop complaining and lamenting about the lack of attention, interest, and action to alleviate infertility where it is desperately needed, and test and use the currently available solutions that can substantially increase access to ART by making diagnosis and treatment affordable without loss of quality. Non-IVF ART treatment options should be the first choice in selected cases, and if IVF-related procedures are required, the combination of inexpensive mild ovarian stimulation protocols and simplified IVF systems will greatly increase access.

Effective and accessible low-cost ART can only be successfully introduced if there is political will and economic and community support. Particularly in LMICs, strengthening infertility services and integrating them with contraceptive and maternal health services within public health structures is essential. Now is the time for action to begin implementation where it is most needed, as it is important to recognize that the choice to have children, even when it requires assistance, is encompassed in the concept of reproductive rights.
